# Differences in Birthweight Outcomes: A Longitudinal Study Based on Siblings

**DOI:** 10.3390/ijerph110606472

**Published:** 2014-06-20

**Authors:** Silvia Bacci, Francesco Bartolucci, Manuela Chiavarini, Liliana Minelli, Luca Pieroni

**Affiliations:** 1 Department of Economics, University of Perugia, Via A. Pascoli, 20, 06123 Perugia, Italy; E-Mail: sbacci@stat.unipg.it; 2 Department of Experimental Medicine, Public Health Section, University of Perugia, P.le Gambuli, 1, 06122 Sant’Andrea delle Fratte, 06156 Perugia, Italy; E-Mails: manuela.chiavarini@unipg.it (M.C.); liliana.minelli@unipg.it (L.M.); 3 Department of Political Sciences, University of Perugia, Via A. Pascoli, 20, 06123 Perugia, Italy; E-Mail: lpieroni@unipg.it

**Keywords:** birthweight, maternal characteristics, standard certificate of live birth

## Abstract

*Objectives*: We investigate the differences in birthweight between first- and second-borns, evaluating the impact of changes in pregnancy (e.g., gestational age), demographic (e.g., age), and social (e.g., education level, marital status) maternal characteristics. *Data and Methods*: All analyses are performed on data collected in Umbria (Italy) taking into account a set of 792 women who delivered twice from 2005 to 2008. Firstly, we use a univariate paired *t*-test for the comparison between weights of first- and second-borns; Secondly, we use linear and nonlinear regression approaches in order to: (i) evaluate the effect of demographic and social maternal characteristics and (ii) predict the odds-ratio of low and high birthweight infants, respectively. *Results*: We find that the birthweight of second-borns is significantly higher than that of first-borns. Statistically significant effects are related with a longer gestational age, an increased number of visits during the pregnancy, and the gender of infants. On the other hand, we do not observe any significant effect related with mother’s age and with other characteristics of interest.

## 1. Introduction

Categorical risk indicators based on birthweight are largely used in perinatal clinics and research. Part of the literature [1,2] investigated the relationship between birthweight and associated mortality rate and suggested the existence of population-specific standards for birthweight. However, the picture of a biological specificity of the birthweight was criticized by some studies in ethnically homogenous populations. Among others, Carlson and Hoem [3] found, in a study concerning the Czech Republic, that differences in birthweight distributions come from underlying differences in lifestyle and social conditions (see also [4,5,6]). Thus, to limit the impact of confounding factors, empirical analyses focus on birth outcomes of siblings. Skjaerven *et al.* [7], for example, found a significant correlation between the birthweight of siblings, even if their study does not evaluate whether the level of association is affected by mothers’ individual and socioeconomic characteristics.

The purpose of the present study is to bridge the lack of the literature mentioned above and to examine differences in birthweight between first- and second-borns, evaluating the impact of changes in pregnancy (e.g., gestational age), demographic (e.g., age), and social (e.g., education level, marital status) maternal characteristics. These estimates are possible by re-examining information available from the Standard Certificate of Live Birth (SCLB) of a population of contemporary Italian women in the Umbria region and selecting a sample of 792 women who delivered twice from 2005 to 2008. Umbria is a region of around 900,000 inhabitants, situated in central Italy, where the number of births per year is around 8,000. Birthrate and fertility indexes have constantly increased and have moved close to the national average (birth-rate 2007: Italy 9.5%, Umbria 8.9%; fertility index 2007: Italy 1.40, Umbria 1.38).

The adopted longitudinal dataset, which reflects the low birth-rate of Italy in comparison to other European countries, also allows us to estimate the tendency toward a repetition of adverse pregnancy outcomes and, indirectly, to underlie the strength of the biological influence on determining birthweight at different occasions. While observational studies report increasing risks of the adverse pregnancy outcomes in low weight babies, there is not much theory to explain the increase of low weight borns during the second pregnancy. An exception is the nutritional depletion hypothesis [8,9]. This suggests that a too short period between pregnancies affects the recovering of the nutritional reserves for supporting fetal growth and contributes to the risk of fetal growth restriction. On the other hand, changes of mother’s behavior in conducting pregnancies (*i.e.*, the reduction of the number of visits), in mother’s clinical patterns (*i.e.*, gestational age), or gender composition of the child may explain differences in birthweights.

Our results show that birthweight of second-borns is significantly higher than that of first-borns. The effects of this difference are related with a longer gestational age, an increased number of visits during the pregnancy, and gender of infants. Instead, we do not observe any significant effect related with the mother’s age and with the other characteristics of interest. Besides, gestational age and gender result to be statistically significant also in explaining low and high weight births in comparison with normal weight babies.

We conclude that the odds-ratio between low birthweight babies *vs.* normal or high birthweight babies, as well as the odds-ratio of low or normal birthweight babies *vs.* high birthweight babies, are more than 2.5 times for each added gestational week and they are 0.24 times for females with respect to males.

The structure of the article is as follows. The next section describes the data and the study population, the variables of interest, and the adopted statistical methods. Section 3 illustrates, from a statistical point of view, the main results obtained through the analyses. To conclude, these results are discussed in Section 4.

## 2. Methods

### 2.1. Data Source and Study Population

The study is based on a set of data obtained from the administration of the Standard Certificate of Live Birth (SCLB) in the Umbria region (Italy) in years 2005–2008. SCLB is filled in within ten days after the delivery by one of the attendants the birth (e.g., doctor, midwife) and it collects information on the infants and mothers. Regarding each mother we know: age, citizenship, educational attainment, marital status, childbearing history, and prenatal care history (e.g., number of visits, gestational age at the first visit). In particular, we use mother’s citizenship to capture the effect on the birthweight of being a foreign mother with respect to being an Italian mother, even if this analysis may lead to an underestimation of this effect since it does not account for mothers born outside but having Italian citizenship. It is also worth noting that we use marital status as a proxy for living in a couple because our dataset does not include mother’s information about cohabitation. Referred to the infant, information include: birthweight, gestational age, and gender. For our study we consider women that delivered for the first time during 2005–2008 and that delivered at least twice in this time interval; more precisely, we take into account information about the first and the second delivery. Moreover, we limit our attention to natural conceptions (*i.e.*, without assisted fertilization methods) and singleton births. Finally, only infants with a gestational age of at least 22 weeks and a birthweight of at least 500 grams are taken into account. The total sample size resulting from the merging of each mother and her baby information amounts to 792.

### 2.2. Variables of Interest

As already mentioned, our main interest is in the analysis of the difference in birthweight between first- and second-borns and the discovery of the significant determinants of this difference. For this aim, we here describe the main variables which are available in the dataset.

Commenting Table 1 and Table 2, reporting some descriptive statistics about the variables of interest, we observe an average birthweight equal to 3276.25 grams for the first babies, that increases to 3365.42 grams for the second babies. Several maternal characteristics may be assumed to explain this difference. We mainly take into account aspects that are strictly related with each pregnancy and, therefore, that can modify from one pregnancy to another one. *In primis*, we refer to the mother’s age, which in our dataset increases in average of two years between the first two deliveries (from 29.44 years for the first pregnancy to 31.51 for the second pregnancy). Moreover, 20.58% of deliveries takes place in less than 18 months apart from each other, whereas the birth intervals exceed 36 months in 12.12% of cases.

**Table 1:** Distribution of variables for first and second newborns: mean and standard deviation for quantitative variables and percentage values for categorical variables.

**Variable**	**Category**	**First newborn**			**Second newborn**		
		**Mean**	**Std. Dev.**	%	**Mean**	**Std. Dev.**	**%**
birthweight (gr.)		3276.25	530.02		3365.42	491.82	
mother’s age (years)		29.44	4.76		31.51	4.85	
gestational age (weeks)		39.32	1.98		39.10	1.74	
time of first visit (gest. weeks)		7.84	4.13		8.20	3.20	
term of delivery	at term			94.15			96.44
	preterm			5.85			3.56
number of visits	sufficient			92.75			91.86
	insufficient			7.25			8.14
course of pregnancy	physiological			96.18			97.20
	pathological			3.82			2.80
gender	male			50.13			53.44
	female			49.87			46.56

**Table 2:** Distribution of differences between second newborns and first newborns: mean and standard deviation for quantitative variables and percentage values for categorical variables

**Variable**	**Category**	**Mean**	**Std. Dev.**	**^%^**
diff. in birthweight (grams)		89.169	532.87	
diff. in mother’s age (years)		2.08	0.67	
diff. in gestational age (weeks)		−0.23	2.19	
diff. in time of first visit (gest. weeks)		0.36	5.01	
birth intervals	less than 18 months			20.58
	between18 and 36 months			67.30
	more than 36 months			12.12
term of deliveries	same type of term			92.30
	at term then preterm			2.78
	preterm then at term			4.92
diff. in number of visits	no difference			87.75
	sufficient then insufficient visits			6.44
	insufficient then sufficient visits			5.81
course of pregnancy	same type of pregnancy			94.69
	physiol. then pathol.			3.16
	pathol. then physiol.			2.15
diff. in gender	same gender			47.73
	male then female			24.49
	female then male			27.78

We also consider possible differences in the pregnancy duration and in the prenatal care. The pregnancy duration is measured in terms of (i) gestational weeks and (ii) preterm deliveries (*i.e.*, deliveries happening before 37 gestational weeks). Second babies tend to be born slightly before than their older siblings (−0.23 gestational weeks), even if preterm deliveries take place in 3.56% of cases *vs.* 5.85% of first babies. In detail, we can observe that 2.78% of women delivers the first born after 37 weeks and the second-born before 37 weeks; on the other hand, 4.92% of women delivers the first born before 37 weeks and the second-born after 37 weeks.

The prenatal care is measured by two proxies: (i) time of the first visit and (ii) total number of prenatal care visits, distinguishing between an insufficient (less than 4) and a sufficient (at least 4) number of visits. The first visit happens slightly later during the second pregnancy (8.20 gestational weeks *vs.* 7.84 gestational weeks for the first pregnancy) and a higher proportion of women tends to reduce the total number of care visits (8.14% *vs.* 7.25% declares an insufficient number of visits). Besides, 6.44% of women has an insufficient number of visits during the second pregnancy although it was sufficient during the first one with respect to 5.81% of women showing an apposite behavior.

Another element that can have a certain influence on birthweight is the course of pregnancy, which is pathological for the 3.82% of first pregnancies and 2.80% of second ones. More in detail, we distinguish between cases of physiological pregnancies followed by pathological pregnancies (3.16%) and pathological pregnancies followed by physiological ones (2.15%).

Concerning gender of infants, in 47.73% of cases the second-born has the same gender than his/her older sibling, in 24.49% of cases a female infant comes after a male one, and in the remaining 27.78% of second pregnancies a male infant comes after a female one.

Finally, as control variables we also include in the analysis some social characteristics of women, such as citizenship, marital status, and educational level (Table 3). We observe that 79.04% of women is Italian, whereas 11.36% comes from East-Europa. In addition, 87.50% of women is married and more than one half (50.76%) has a high school diploma, followed by 28.91% with a higher educational level (degree or above); the remaining 20.33% of women attained at most a compulsory educational level.

**Table 3:** Distribution of control variables.

**Variable**	**Category**	**%**
citizenship	Italian	79.04
	East-Europe	11.36
	other citizenship	9.60
marital status	married	87.50
	not married	12.50
educational level	middle school or less	20.33
	high school	50.76
	degree or above	28.91

### 2.3. Statistical Methods

We first test that the difference in birthweight between first- and second-borns is equal to 0 by a univariate paired *t*-test. Secondly, we consider the difference in birthweights as a quantitative response variable in a linear regression model, where the explanatory variables previously described are introduced as covariates. Finally, the birthweight of first- and second-borns is categorized in a suitable way and the longitudinal structure of data is explicitly taken into account in a conditional (or fixed effects) ordered logit model [10,11,12,13,14]. In detail, we define a categorical variable for the birthweight, which corresponds to a low birthweight (<2,500 gr), normal weight (between 2,500 and 4,000 gr), and high birthweight (≥4,000 gr), as suggested by the majority of the literature [15]. The proposed model is based on cumulative logits [16,17] which compare normal or high birthweight category *vs.* low birthweight category and high birthweight category (*i.e.*, macrosomic infants) *vs.* low or normal birthweight category. We also take into account the same covariates considered in the linear regression analysis, so as to obtain a useful model for predicting possible negative outcomes in terms of low and high birthweight due to the effect of statistically significant time-varying covariates.

## 3. Results

As illustrated in Table 4, reporting the results of the paired *t*-test, second-borns tend to have a birthweight greater than 89.17 grams with respect to the first borns. With a *p*-value smaller than 0.001, the test allows us to conclude for a substantially significant difference between the two outcomes.

**Table 4:** Paired *t*-test for the comparison between birthweights.

**Variable**	**Value**	**Std. err.**	***t*-value**	**df**	***p*-value**
difference in birthweight	89.17	18.93	4.71	791	0.000

In order to detect possible determinants of the difference at issue, a linear regression model is estimated taking into account the covariates described in Table 2 and Table 3. The parameter estimates of the model containing all the possible covariates are shown in Table 5, whereas those of the model obtained by a backward selection process and containing only the statistically significant covariates are reported in Table 6. These tables show the following quantities: estimates of regression coefficients, corresponding standard errors, *p*-values, and the inferior and superior limits (denoted by *l*_1_ and *l*_2_, respectively) of the 95% confidence intervals.

The proposed model (Table 5) explains 41.86% of the global variance of the response variable; however, we observe that most covariates are not statistically significant. More precisely, no effect may be ascribed to the increasing of the mother’s age nor to the time of the first prenatal care visit. Neither the birth intervals help to explain the general tendency to the increase of birthweight. We only observe a certain effect (*p*-value = 0.088) related with possible changes in the course of the second pregnancy with respect to the first one: a physiological pregnancy that follows to a pathological one results in a positive difference of birthweight (+151.43 grams) rather than second pregnancies having the same course of the first ones. On the other hand, no significant difference is observed in case of pathological pregnancies following physiological pregnancies.

**Table 5:** Linear regression results for the differences in birthweight: all covariates (adjusted *R*^2^ = 0.4186).

**Variable**	**Category**	**est.**	**std. err.**	***p*-value**	***l*_1_**	***l*_2_**
constant		9.01	96.35	0.926	−180.12	198.15
diff. in mother’s age		39.80	40.40	0.325	−39.52	119.11
diff. in gestational age		136.57	8.55	0.000	119.79	153.34
diff. in time of first visit		−1.92	2.96	0.516	−7.73	3.89
birth intervals	bet. 18 and 36 months					
	less than 18 months	46.48	50.80	0.361	−53.25	146.21
	more than 36 months	−15.48	64.83	0.811	−142.75	111.78
term of deliveries	same type of term					
	at term then preterm	−212.09	97.51	0.030	−403.51	−20.67
	preterm then at term	154.24	79.98	0.054	−2.76	311.25
diff. in number of visits	no difference					
	suffic. then insuffic. visits	−53.71	61.58	0.383	−174.60	67.18
	insuffic. then suffic. visits	102.31	63.13	0.106	−21.62	226.23
course of pregnancy	same type					
	physiol. then pathol.	−132.76	101.72	0.192	−332.44	66.93
	pathol. then physiol.	151.43	88.59	0.088	−22.48	325.34
diff. in gender	same gender					
	male, then female	−108.52	36.38	0.003	−179.93	−37.12
	female, then male	135.20	34.82	0.000	66.85	203.55
citizenship	Italian					
	east-europe	12.13	48.90	0.804	−83.85	108.12
	other citizen.	92.49	52.97	0.081	−11.49	196.47
marital status	married					
	not married	11.37	44.18	0.797	−75.35	98.10
educational level	middle school or less					
	high school	−11.51	40.40	0.776	−90.81	67.79
	degree or above	−4.42	45.35	0.922	−93.44	84.61

Concerning mother’s social characteristics, we only observe a weak effect of citizenship (*p*-value = 0.081): second-borns of women of other citizenship tend to present a greater birthweight increase with respect to those of Italian women. This can be explained by a greater awareness of the possibility and usefulness of prenatal care that foreign women gain during the first pregnancy, whereas Italian women are generally well-informed already from the beginning of first pregnancy.

On the basis of the model resulting by the backward selection process (Table 6), we may impute a large part (41.94%) of the variability of the analyzed phenomenon to the effect of: (i) differences in gestational ages; (ii) differences in term of deliveries; (iii) differences in the number of prenatal care visits between first and second delivery; and (iv) differences in the gender of first- and second-born babies.

**Table 6:** Linear regression results for the differences in birthweight: statistically significant covariates (α = 0.05; adjusted *R*^2^ = 0.4194).

**Variable**	**Category**	**est.**	**std. err.**	***p*-value**	***l*_1_**	***l*_2_**
constant		97.84	21.85	0.000	54.95	140.74
diff. in gestational age		139.83	8.33	0.000	123.47	156.19
term of deliveries	same type of term					
	at term, then preterm	−196.25	96.23	0.042	−385.15	−7.34
	preterm, then at term	179.65	78.25	0.022	26.05	333.26
diff. in number of visits	no difference					
	insuffic. then suffic. visits	130.87	61.80	0.035	9.55	252.19
diff. in gender	same gender					
	male, then female	−104.92	35.92	0.004	−175.42	−34.41
	female, then male	136.74	34.5	0.000	69.01	204.47

Each additional gestational week leads to an average increase of 139.83 grams in the birthweight (*l*_1_ = 123.47*, l*_2_ = 156.19). Moreover, preterm deliveries that follow at term deliveries are associated with a birthweight smaller than same term deliveries (−196.25 gr) and, in similar way, at term deliveries following preterm deliveries present a higher birthweight increase (+179.65 gr) than same term deliveries. Another partly significant effect is due to the number of prenatal care visits: second-borns with a sufficient number of visits during the pregnancy show an increase of 130.87 grams (*l*_1_ = 9.55*, l*_2_ = 252.19) with respect to their sibling unsatisfactorily followed during the previous pregnancy. We also observe that the effects of the term of deliveries and of the number of prenatal care visits are both significant at 5% level, but not at 1% level.

Finally, the different gender between siblings plays a relevant role. In the presence of first male borns and second female borns, we may observe a negative difference between the birthweights (−104.92 gr) with respect to the case of siblings having the same gender. Similarly, we observe a positive difference (+136.74 gr) in the case of first female borns and second male borns.

To conclude, we outline that the main part of the variability of the differences in birthweight between first- and second-borns is not explainable through the independent variables taken into account in our analysis. Indeed, after controlling for all the significant covariates, it remains an expected residual increase in birthweight equal to 97.84 gr (*p*-value < 0.0001; *l*_1_ = 54.95*, l*_2_ = 140.74). In other words, a woman with a second pregnancy equal to the first one for gestational age, term of delivery, number of prenatal care visits, and gender of baby can expect an infant with a weight of 97.84 grams higher than the younger sibling. One or more changes in the mentioned significant variables give further changes in the birthweight, according with the estimated regression coefficients of Table 6.

As mentioned at the end of Section 2, it may be useful to develop a model for the prediction of negative outcomes in second babies, given information about first babies. In this regard, Table 7 shows the conditional distribution of the birthweight of second-borns (birthweight_2_) given the birthweight of first-borns (birthweight_1_). On one hand, the frequency of low birthweight second babies given normal weight first babies is equal to 2.9%; however, this value rises to 20.5% when first babies have low birthweight. Similarly, the proportion of high birthweight second babies given normal weight first babies is equal to 6.6%; however, this value rises to 27.3% when first babies have high birthweight, too.

**Table 7:** Absolute frequencies of low, normal and high weights of first and second babies (in parentheses there are the conditional percentage values of second newborns given first newborns).

**Birthweight_1_**	**Birthweight_2_**			
	**Low birthweight**	**Normal weight**	**High birthweight**	**Total**
low birthweight	9 (20.5)	34 (77.3)	1 (2.3)	44 (100.0)
normal weight	20 (2.9)	627 (90.5)	46 (6.6)	693 (100.0)
high birthweight	0 (0.0)	40 (72.7)	15 (27.3)	55 (100.0)
Total	29 (3.7)	701 (88.5)	62 (7.8)	792 (100.0)

The tendency to have a low or a high birthweight baby may be predicted through a conditional ordered logit model for longitudinal data, where the categorical birthweight of first- and second-borns is taken as response variable and the other variables listed in Table 5 are included as covariates. We outline that the adopted model is estimated by the conditional maximum likelihood method (for a review, see [21]). As a main consequence, only the time-varying covariates may be estimated, whereas the time-constant covariates (such as citizenship) are dropped from the analysis. Results in Table 8 show that gestational age and gender are the only significant covariates which can explain differences in the probability of having babies with different birthweight. On one hand, we may conclude that for each additional gestational week the odds-ratio of having a normal or high birthweight baby increases 2.61 times as well as the odds-ratio of having a high weight baby; the same odds-ratio reduces by 76% (odds-ratio estimate equals 0.24) for females with respect to males. On the other hand, as concerns the remaining covariates, a certain effect (*p*-value = 0.076) is due to the course of pregnancy, whereas the intervention covariates, such as time of first prenatal care visit and number of visits, do not play any significant role for the birthweight prediction.

**Table 8:** Conditional ordered logit regression results for the birthweight: all covariates *.

**Variable**	**Category**	**est.**	**std. err.**	***p*-value**	***l*_1_**	***l*_2_**	**odds-ratio**
mother’s age		0.00	0.00	0.067	0.00	0.00	1.00
gestational age		0.96	0.17	0.000	0.63	1.29	2.61
time of first visit		0.03	0.06	0.636	−0.09	0.15	1.03
number of visits	insufficient						
	sufficient	0.61	0.78	0.436	−0.92	2.14	1.84
course of pregnancy	physiological						
	pathological	−2.07	1.16	0.076	−4.35	0.22	0.13
gender	male						
	female	−1.41	0.34	0.000	−2.08	−0.75	0.24

* Estimated odds-ratios refer to normal or high birthweight infants *vs.* low birthweight infants and to high birthweight infants *vs.* low or normal birthweight infants.

## 4. Conclusions

We find that the birthweight of second-born infants is on average 89 grams higher than the birthweight of the younger siblings. Significant determinants are obtained for differences in gestational age, deliveries term, number of prenatal visits, and in gender.

The association between gestational age and birthweight (and mortality) is one of the most studied topic within perinatal epidemiology [18]. Observational studies showed unambiguously that the firstand second-born birthweights depend on the specific distribution of mothers’ delivery term [19]. The potential determinants summarized by the weight-specific fetal growth rate curves indicate the gestational age as the main explanatory variable affecting the weight of newborns, in particular when associated with pathological pregnancies.

Although the birthweight is partly explained by a genetic influence [20], a positive correlation between terms of delivery and birthweight is confirmed in the change from preterm deliveries to at term deliveries or vice-versa. There is a remarkable difference on birthweight if this clinical determinant arises and the underlying mechanism is similar to that for the gestational age. There is an increase in the risk for a preterm birth (and a low birthweight) when an abnormal fetal growth pattern is recognized.

Varying the suggested number of visits from insufficient to sufficient seems to explain the weight increase of the second infant. A possible explanation is that these infants benefit from their linkage to a socioeconomic level and individual characteristics by the reduction of access inequality to prenatal care, for which the number of visits represents the most important indicator (see, for example, [21]). In particular, previous studies showed that occupational status [22,23] and being unmarried [24,25,26,27] are all barriers to early initiation of prenatal care and execution of an appropriate number of prenatal visits. These factors may account for changes in the immigration status which may lead to greater use of the prenatal access [28]. For example, prenatal care utilization may have had a shift in Italy from the European enlargement in 2007, which gave to the East-European women the possibility to extend their permits to stay in Italy or, whether irregular immigrants, the opportunity to regularize their status.This hypothesis is confirmed by Chiavarini *et al.* [29], who found for the same region of Umbria a reduction of the inequality of the prenatal care utilization of the 30% from 2005 to 2010.

Differences in second birthweight by changes in gender (e.g., positive from female to male; negative from male to female) might be explained by the specific intrauterine growth patterns. As argued by Wald *et al.* [30] and Catalano *et al.* [31], the reasons for these sex-related differences are still unclear even if fetal sex seems to affect genetic and environmental regulators of fetal growth [32]. Lampl *et al.* [33] found in a longitudinal study that the growth of male fetuses is more sensitive to maternal weight and height, varying with gestational age. Thus, the authors suggested that fetal sex may regulate the effects of biological and non-biological determinants of intrauterine growth.

Being focused on the average of the weight distribution, the variation of birthweight between first and second birth may have limited implications because it does not refer to high-risk infants [34]. When we focus on the difference of birthweight, we find that the weight of the second-born is predicted significantly from the weight of the first-born, whereas the number of prenatal care visits and the time of the first visit are not predictors of babies at risk of low and high birthweights.

Our results suggest that factors affecting the probability of having a low or high birthweight first-born also affect the probability of having a second-born baby with problematic birthweight. While we are able to show that the folate depletion hypothesis tested by Smits *et al.* [35] is not supported by the data, as the interval between pregnancies is never significant, we have to conclude cautiously about the public health implications. This statement is also strengthened by some limitations of our sample. On one hand, the decision to give birth twice within a 4-year period may be associated with other birth outcome factors related to the mother’s career, educational level, and wage. The use of sibling fixed effects only partly alleviates this selection concern by focusing strictly on within family differences. On the other hand, the absence in the dataset of some covariates that the current literature has found to affect the birthweight, as for example if a mother smokes or not, might make some estimates questionable. For instance, mothers who smoke during pregnancy have babies that weigh less by 100–200 grams [36]. In addition, we find that, while prenatal care visits are of importance for birthweight distributions, they do not affect the risk of having a baby with risky birthweight.

Finally, we expect to rely on followup studies which account for changes in risky behavioral habits of mothers between pregnancies. Concerning this point, SCLBs have recently been enriched with information about smoking and drinking habits, which can be used in a future development of our work to investigate more thoroughly the determinants of changes in birthweight of subsequent births.

## References

[1] Wilcox A., Russell I (1983). Birthweight and perinatal mortality: I. On the frequency distribution of birthweight. Int. J. Epidemiol.

[2] Wilcox A., Russell I (1990). Why small black infants have a lower mortality rate than small white infants: The case for population-specific standards for birth weight. J. Pediatr.

[3] Carlson E., Hoem J (1999). Low-weight neonatal survival paradox in the Czech Republic. Am. J. Epidemiol.

[4] Shoham-Yakubovich I., Barell V (1988). Maternal education as a modifier of the association between low birthweight and infant mortality. Int. J. Epidemiol.

[5] Foster H.J. (1997). The enigma of low birth weight and race. N. Engl. J. Med.

[6] Chiavarini M., Bartolucci F., Gili A., Pieroni L., Minelli L (2012). Effects of individual and social factors on preterm birth and low birth weight: An Italian case study. Int. J. Public Health.

[7] Skjaerven R., Wilcox A., Russell D (1988). Birthweight and perinatal mortality of second births conditional on weight of the first. Int. J. Epidemiol.

[8] Winkvist A., Rasmussen K., Habicht J (1992). A new definition of maternal depletion syndrome. Am. J. Public Health.

[9] King J (2003). The risk of maternal nutritional depletion and poor outcomes increases in early or closely spaced pregnancies. J. Nutr.

[10] Diggle P.J., Heagerty P., Liang K.Y., Zeger S.L. (2002). Analysis of Longitudinal Data.

[11] Andersen E.B. (1970). Asymptotic properties of conditional maximum-likelihood estimators. J. Royal Stat. Soc. Ser. B.

[12] Andersen E.B. (1972). The numerical solution of a set of conditional estimation equations. J. Royal Stat. Soc. Ser. B.

[13] Chamberlain G (1980). Analysis of covariance with qualitative data. Rev. Econ. Stud.

[14] Baetschmann G., Staub K.E., Winkelmann R (2011). Consistent estimation of the fixed effects ordered logit model. IZA Discussion Paper.

[15] W.H.O. (1975). International classification of disease. Geneva.

[16] McCullagh P (1980). Regression models for ordinal data. J. Royal Stat. Soc. Ser. B.

[17] Agresti A (2002). Categorical Data Analysis.

[18] Melve K., Skjaerven R., Gjessing H., Oyen N (1999). Recurrence of gestational age in sibships: implications for perinatal mortality. Am. J. Epidemiol.

[19] Skjaerven R., Gjessing H., Bakketeig L (2000). Birthweight by gestational age in Norway. Acta Obstet. Gynecol. Scand.

[20] Skjaerven R., Gjessing H., Bakketeig L (2000). New standards for birth weight by gestational age using family data. Am. J. Obstet. Gynecol.

[21] EURO-PERISTAT (2008). www.europeristat.com/.

[22] Johnson A., Hatcher B., El-Khorazaty M., Milligan R., Bhaskar B., Rodan M. (2007). Determinants of inadequate prenatal care utilization by African American women. J. Health Care Poor Underserved.

[23] Beeckman K., Louckx F., Putman K (2011). Predisposing, enabling and pregnancy-related determinants of late initiation of prenatal care. Matern Child Health J.

[24] Braveman P., Bennett T., Lewis C., Egerter S., Showstack J (1993). Access to prenatal care following major medicaid eligibility expansions. JAMA.

[25] Rowe R., Magee H., Quigley M., Heron P., Askham J., Brocklehurst P (2008). Social and ethnic differences in attendance for antenatal care in England. Public Health.

[26] Delvaux T., Buekens P., Godin I., Boutsen M (2001). Barriers to prenatal care in Europe. Am. J. Prev. Med.

[27] Ayoola A., Nettleman M., Stommel M., Canady R (2010). Time of pregnancy recognition and prenatal care use: a population-based study in the United States. Birth.

[28] Lariccia F., Mussino E., Pinnelli A., Prati S (2013). Differences between Italian and foreign women in the use of antenatal care. Genus.

[29] Chiavarini M., Salmasi L., Pieroni L., Lanari D., Minelli L (2013). Access equality to prenatal care in Italy: the effects of socio-demographic determinants. Eur. J. Public Health.

[30] Wald N., Cuckle H., Nanchahal K., Turnbull A (1986). Sex differences in fetal size early in pregnancy. Br. Med. J.

[31] Catalano P., Drago N., Amini S (1995). Factors affecting fetal growth and body composition. Am. J. Obstet. Gynecol.

[32] de Zegher F., Francois I., Boehmer A., Saggese G., Müller J., Hiort O. (1998). Androgens and fetal growth. Horm Res.

[33] Lampl M., Gotsch F., Kusanovic J., Gomez R., Nien J., Frongillo E. (2010). Sex differences in fetal growth responses to maternal height and weight. Am. J. Hum. Biol.

[34] Reeves S., Bernstein I (2008). Optimal Growth Modeling. Semin Perinatol.

[35] Smits L., Essed G (2001). Short interpregnancy intervals and unfavourable pregnancy outcome: role of folate depletion. Lancet.

[36] Abrevaya J., Dahl C.M. (2008). The Effects of Birth Inputs on Birthweight. J. Bus. Econ. Stat.

